# Cuproptosis-Related LncRNA Signature for Predicting Prognosis of Hepatocellular Carcinoma: A Comprehensive Analysis

**DOI:** 10.1155/2022/3265212

**Published:** 2022-11-21

**Authors:** Qiqi Chen, Tong Sun, Guorong Wang, Mengyu Zhang, Yitian Zhu, Xiaonan Shi, Zhishan Ding

**Affiliations:** ^1^The Second School of Clinical Medicine, Zhejiang Chinese Medical University, Hangzhou 310053, China; ^2^School of Medical Technology and Information Engineering, Zhejiang Chinese Medical University, Hangzhou 310053, China; ^3^Hangzhou Medical College, Hangzhou 310053, China

## Abstract

Hepatocellular carcinoma (HCC) is one of the most common malignant tumors worldwide and has a poor prognosis. Cuproptosis is a novel mode of cell death that has only recently been discovered. Considering the critical role of lncRNAs in liver cancer development, the aim of this study was to construct a prognostic signature based on cuproptosis-related lncRNAs (CRlncRNAs). We downloaded RNA-sequencing data and corresponding clinical information of patients with HCC from The Cancer Genome Atlas (TCGA) database. To verify the robustness of the model, we added an external validation set obtained from the Gene Expression Omnibus (GEO): GSE40144. In addition, we identified the cuproptosis-related genes (CRGs) based on previous reports. Pearson correlation analysis, univariate Cox regression, and least absolute shrinkage and selection operator (LASSO) Cox regression analysis were utilized to screen for genes associated with prognosis. On this basis, multivariate Cox regression and stepAIC were used to further construct and optimize the prognostic model. The simplified signature with the lowest Akaike information criterion (AIC) value was considered the prognostic signature. Seven different algorithms were used to perform immune infiltration analysis. The single-sample Gene Set Enrichment Analysis (ssGSEA) algorithm was utilized to find the difference in immune function between the high- and low-risk groups. Finally, in vitro experiments were performed by quantitative real-time PCR (qRT–PCR) analysis using HCC cell lines to validate the expression of prognostic genes. We identified 3 lncRNAs (CYTOR, LINC00205, and LINC01184) as independent risk factors for HCC. The receiver operating characteristic (ROC) curves calculated that the AUC at 1, 3, and 5 years reached 0.717, 0.633, and 0.607, respectively. The expression levels of 41 immune checkpoints differed significantly between the high- and low-risk groups, and there were significant differences in sensitivity to immunotherapy between the high- and low-risk groups. The risk model could also serve as a promising predictor of immunotherapeutic response, which has been verified by the TIDE algorithm (*p* < 0.001). Overall, we propose a signature related to CRlncRNAs that can be used to predict the prognosis of HCC patients, which was validated in external cohort and in vitro experiments.

## 1. Introduction

A recent study proposed a novel mode of cell death, cuproptosis, which occurs by combining copper directly with the lipid components of the tricarboxylic acid (TCA) cycle, resulting in aggregation of lipid proteins and subsequent loss of iron-sulfur cluster proteins, triggering protein toxicity stress, and eventually leading to cell death [1]. Copper plays an important role in many life processes in eukaryotes, such as energy metabolism, reactive oxygen species detoxification, iron absorption, and signal transduction [2]. Previous studies have shown that the toxicity originating from the over intake of copper is an important cause of oxidative damage when copper homeostasis is destroyed [3]. The potent redox activity of copper enables copper to play a unique role as a key regulator of cellular signaling pathways [4]. Increasing evidence shows that serum copper levels are inseparable from HCC proliferation and metastasis, making it an important biomarker of liver cancer [5].

Long noncoding RNAs (lncRNAs), which are defined as transcripts longer than 200 nucleotides with no protein coding potential, many of which are distinctively expressed in specific tissue or cancer types, play an important role in the development of cancer [6, 7]. The remodeling of the tumor microenvironment and tumor immune escape is inseparable from the changes in metabolic activities mediated by lncRNAs [8]. A large number of cancer-associated lncRNAs have been implicated in the regulation of cancer invasion and metastasis [9, 10]. LOXL1-AS1 drives HCC cell proliferation and migration by regulating the miR-377-3p/NFIB axis [11]. High expression of NKILA advances liver cancer cell proliferation, invasion, and EMT by targeting miR-485-5p [12]. The miR-326/hnRNPA2B1 axis is regulated by PCAT6 to promote cancer cell proliferation and increase its invasiveness [13]. On the other hand, lncRNAs can also silence cancer cells [14, 15].

HCC is a malignant tumor with poor prognosis and is one of the leading causes of cancer-related deaths worldwide [16]. Viral hepatitis, smoking, obesity, fatty liver disease, etc. are considered risk factors for HCC [17]. The scope of surgical resection is only suitable for early-stage patients, while the proportion is less than 15% [18]. Against the backdrop of major advances in medical management, the prognosis for HCC patients remains poor, posing significant conundrums for clinical therapists [19], and the discovery of new and effective prognostic biomarkers for HCC is particularly important.

## 2. Materials and Methods

### 2.1. Data Sources

The RNA-seq transcriptome data derived from 374 tumor samples and 50 adjacent normal tissues and corresponding clinical data of patients with LIHC were downloaded from TCGA (https://portal.gdc.cancer.gov/) database and normalized by transcripts per million (TPM). A list of CRGs (FDX1, CDKN2A, DLD, DLAT, LIAS, GLS, LIPT1, MTF1, PDHA1, and PDHB) was retrieved from Tsvetkov's publication [1]. To verify the robustness of the signature, we collected 59 patients' information from the GEO (GSE40144, https://www.ncbi.nlm.nih.gov/geo/). Samples without complete survival data were excluded. Coexpression analysis of CRGs and lncRNAs was performed using Pearson's correlation, and the association was considered significant if the correlation coefficient |*R*^2^| > 0 at *p* < 0.001. The basic information of the TCGA-LIHC and GSE40144 was summarized in Table 1.

### 2.2. Construction and Validation of a CRlncRNAs Prognostic Signature

Univariate Cox regression analysis was performed to screen CRlncRNAs significantly associated with overall survival (OS) in the TCGA-LIHC dataset. The TCGA-LIHC dataset was randomly divided into a training set (*n* = 185) and a testing set (*n* = 185). We performed LASSO Cox regression analysis in the training set, and the R package “glmnet” was used to identify prognosis-related genes by 1000-fold cross validation. The obtained genes were included in multivariate Cox regression analysis to establish the prognostic signature based on the lowest AIC value in the training set, while testing set and total set were used to validate the signature. The risk score for each patient was calculated using the following formula:

(1) $$\begin{array}{c} Risk\;Score=\overset{n}{\underset{i=1}{\sum }}\left({\text{Exp}}_{i}*{Coe}_{i}\right). \end{array}$$

Patients were divided into high- and low-risk groups based on the median value of the risk score. Currently, ROC curves are widely used for the validation of prediction models in biological and medical related fields [20–22]. In this study, ROC curve was drawn using the “timeROC” package. Moreover, Kaplan–Meier survival analysis was then conducted to display the prognostic performance of the signature, which was performed using the “survival” and “survminer” packages.

### 2.3. Nomogram

We applied the “rms” package to construct the nomogram, aiming to predict the 1-, 3-, and 5-year OS rates of HCC patients by using the patient's risk score and clinical characteristic information. Calibration plots were used to test the predictive power of the nomogram.

### 2.4. Immunity Landscape Assessment

Seven algorithms (TIMER, CIBERSORT, CIBERSORT—ABS, QUANTISEQ, MCPCOUNTER, XCELL, and EPIC) were utilized to analyze the immune microenvironment of tumors. ssGSEA was used to further quantify the enrichment levels of immune cells and immune function with the aim of evaluating immunological signatures between the high- and low-risk groups. Meanwhile, to validate the predictive power of the signature on anti-PD1 and anti-CTLA4 response, we downloaded data from TIDE (http://tide.dfci.harvard.edu/), and we visualized the results using the “ggpubr” package.

### 2.5. Analysis of Tumor Mutational Burden in Different Risk Groups

Tumor mutational burden (TMB), defined as nonsynonymous somatic mutations per megabase in the coding region, was counted by the total number of mutations [23]. We used the *read.maf* function to read the MAF file, and the TMB levels of each patient in the MAF file was calculated using the “maftools” package. The top 15 genes with the highest mutation frequency in TCGA-LIHC cohort were analyzed in both high- and low-risk groups.

### 2.6. Statistical Analysis

All statistical analyses in the study were performed using R software (version 4.1.3). Principal component analysis (PCA) was used to explore whether the high- and low-risk groups were distributed in the discrete direction, and the results were visualized using the “scatterplot3D” package in R software. The package “pec” was applied to C-Index analysis. We utilized “corrplot” to perform gene correlation analysis. Gene Set Enrichment Analysis was performed using “http://org.Hs.eg.db”, “DOSE”, “clusterProfiler” and “enrichplot” packages. Student's *t* test was used for clinical correlation analysis. The statistical significance threshold was set at *p* < 0.05 unless otherwise stated.

### 2.7. Cell Culture

Human HCC cell lines (HepG2) and human hepatic epithelial cells (LO2) were purchased from the National Certified Cell Culture Collection Center (Shanghai, China) and cultured in high-glucose DMEM containing 10% fetal bovine serum (DMEM, Gibco, China). Cell culture was performed in a cell incubator at 37°C and 5% carbon dioxide.

### 2.8. RNA Extraction and qRT–PCR

Total cellular RNA was extracted using TRIzol reagent (YISHAN Bio, Shanghai, RN001) according to the manufacturer's protocol. cDNA synthesis was reverse transcribed using the PrimeScript RT kit (BeyoRTII, China). Data were collected during each extension phase of PCR and analyzed using a StepOnePlus Real-Time PCR instrument (Applied Biosystems, USA). Human GAPDH was selected as an endogenous control (Sangon, Shanghai, B661104-0001). We repeated the process three times for each sample, and the relative quantification of lncRNAs was calculated using the 2-*ΔΔ*CT method. GraphPad Prism (version 8.0) was used to draw graphs. The sequences of all primers used in this study were provided in Table 2.

## 3. Result

### 3.1. Identification of CRlncRNAs

**Figure 1:**
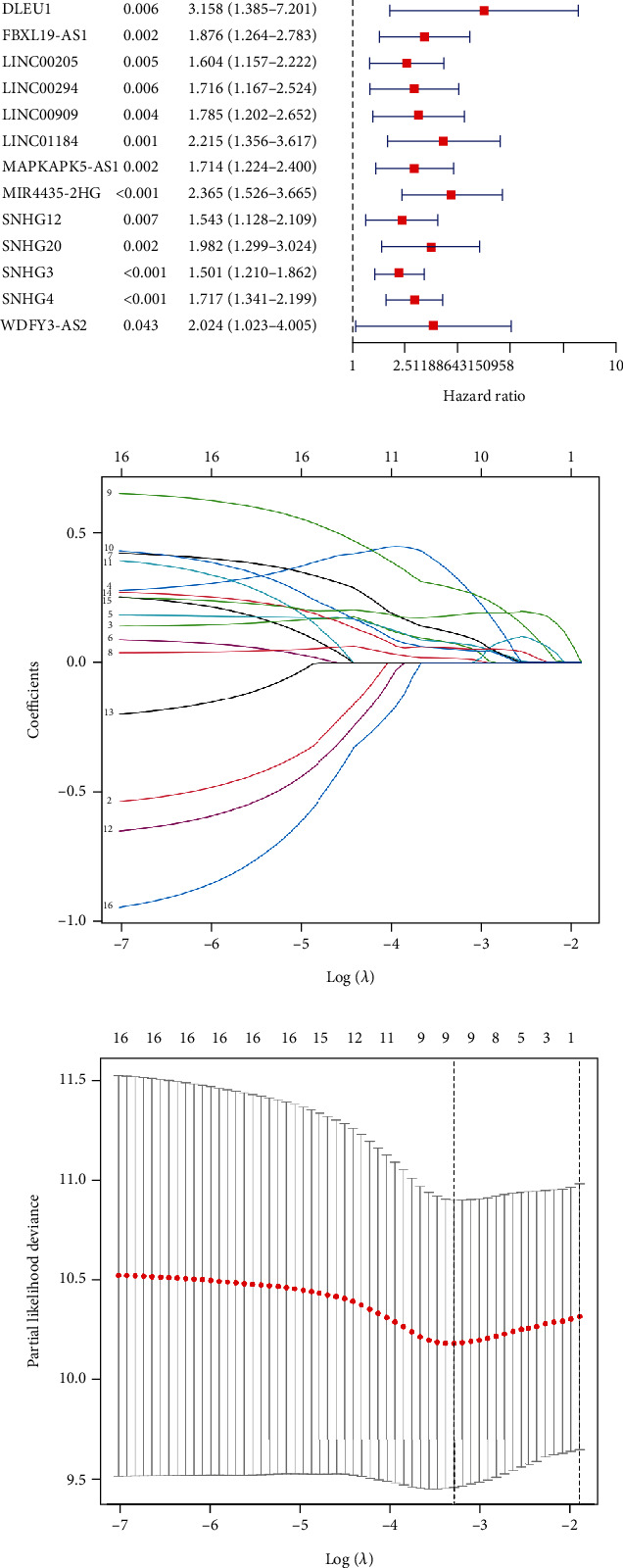
Identification of prognosis-related CRlncRNAs in TCGA cohort. (a) Forest plot of univariate Cox regression identified 16 CRlncRNAs significantly correlated with OS of HCC patients. (b, c) LASSO regression screened of cuproptosis-related lncRNAs at the minimum point of cross-validation.

We evaluated the prognostic relationship between CRlncRNAs expression and OS in the TCGA-LIHC cohort by univariate Cox regression and found 16 CRlncRNAs (*p* < 0.05, Figure 1(a)). To avoid overfitting, we incorporated the above 16 prognosis-related CRlncRNAs into the LASSO regression and obtained 9 candidates lncRNAs (Figures 1(b) and 1(c)).

### 3.2. Establishment and Evaluation of Prognostic Signature

Next, we conducted an in-depth analysis of the prognosis-related lncRNAs using multivariate Cox regression and found 3 lncRNAs (CYTOR, LINC00205, and LINC01184) that were strongly associated with the prognosis of HCC patients. Based on the expression levels of the 3 lncRNAs and the corresponding weighted coefficients, we constructed a prognostic signature for HCC patients: Risk Score = 0.2989 × Exp (CYTOR) + 0.5550 × Exp (LINC00205) + 0.4254 × Exp (LINC01184).

**Figure 2:**
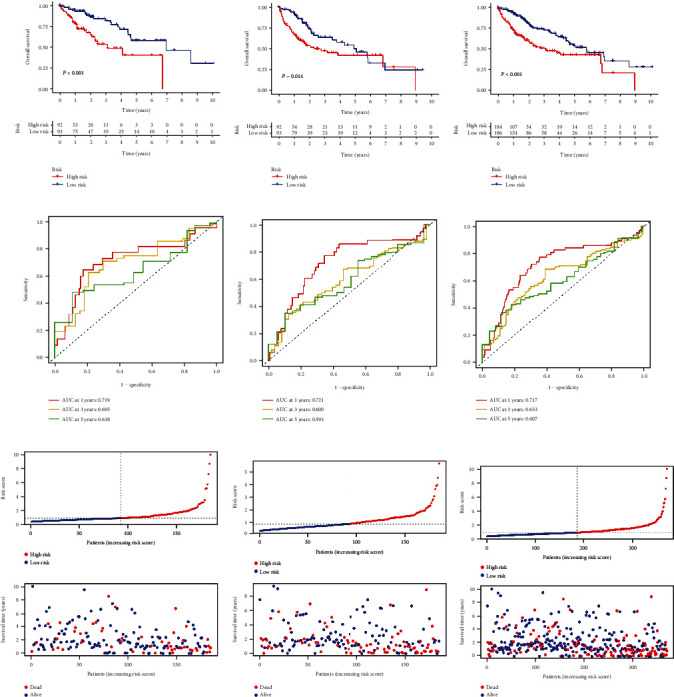
Development and validation of the 3-gene signature in TCGA cohort. (a–c) K-M survival analysis of training set, testing set and total set. (d–f) ROC curves predicted 1-, 3-, and 5-year OS for training set, testing set, and total set. (g–i) Risk scores and survival status plots of training set, testing set, and total set.

We substituted the expression information of related genes of HCC patients into the above prognostic formula to calculate the risk score. The division of high- and low-risk groups for the testing set and total set was based on the median risk value of the training set. We performed a differential analysis of clinical traits between the three data sets. Using Kaplan–Meier analysis, we found that in all three data sets, the high-risk group had significantly lower OS than the low-risk group (*p* < 0.001) (Figures 2(a)–2(c)). ROC curve was used to evaluate the accuracy of the prognostic signature, demonstrating that CRlncRNAs have excellent and robust predictive ability in the training set (1 − year AUC = 0.719, 3 − year AUC = 0.695, 5 − year AUC = 0.638; Figure 2(d)), testing set (1 − year AUC = 0.721, 3 − year AUC = 0.600, 5 − year AUC = 0.593; Figure 2(e)) and total set (1 − year AUC = 0.717, 3 − year AUC = 0.633, 5 − year AUC = 0.607; Figure 2(f)).

**Figure 3:**
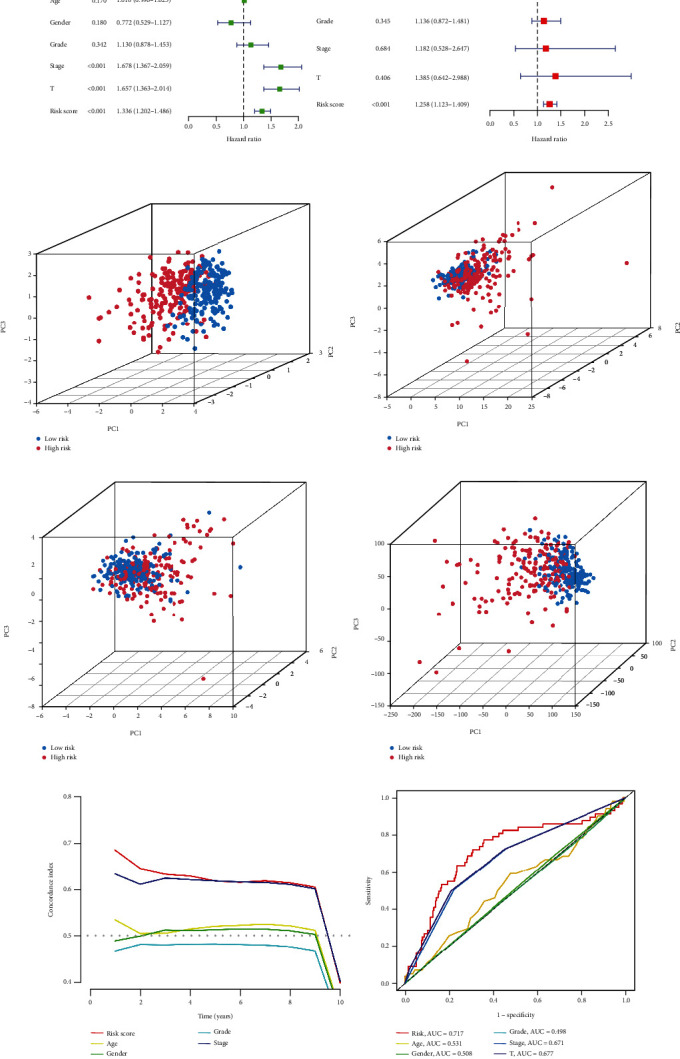
A series of assessments of the predictive ability of prognostic signature. (a, b) Univariate and multivariate Cox regression analyses of clinical information and risk score. PCA observed the distribution of high- and low-risk patients according to (c) risk lncRNAs, (d) cuproptosis-related lncRNAs, (e) cuproptosis related genes and (f) all genes. (g) The C-index curve between the high-risk group and low-risk group in the TCGA cohort. (h) ROC curve of different clinical characteristics.

**Figure 4:**
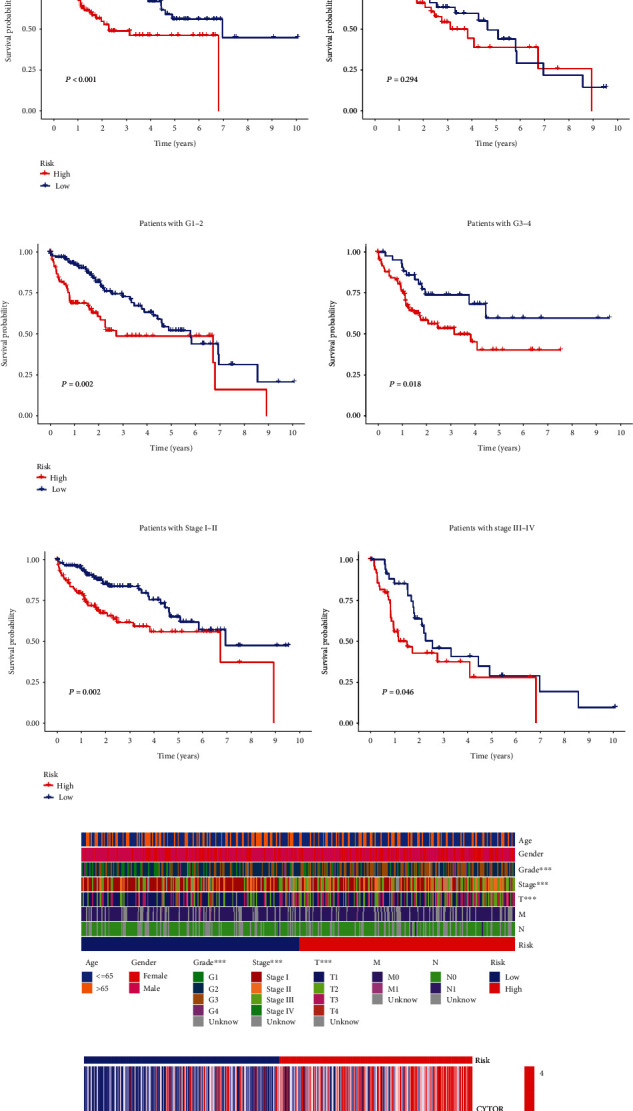
Clinical correlation analysis in TCGA cohort. K-M survival analysis for clinicopathological subgroups divided by (a) age, (b) sex, (c) grade, and (d) AJCC stage. (e) Clinical correlation heat map. (f) Expression heat map of 3cuproptosis-associated lncRNAs. ^∗^*p* < 0.05, ^∗∗^p < 0.01, ^∗∗∗^p < 0.001.

We also established a patient risk-survival status plot (Figures 2(g)–2(i)). Univariate and multivariate Cox analyses revealed that age and risk score were independent prognostic factors for HCC patients (Figures 3(a) and 3(b)). PCA indicated that HCC patients in different risk groups were distributed in two directions and the lncRNAs involved in signature construction had the best discrimination (Figures 3(c)–3(f)). The C-index curve (Figure 3(g)) and ROC curve (Figure 3(h)) showed that the prognostic signature had the best predictive ability (AUC = 0.717) compared with age, sex, grade, and AJCC stage. We also carried out signature validation of clinical grouping for four indicators of interest (age, AJCC stage, and grade), and the results showed that the signature was suitable for different ages (Figure 4(a)), sexes (Figure 4(b)), grades (Figure 4(c)), and AJCC stages (Figure 4(d)).

### 3.3. Clinical Correlation Analysis

We further explored the difference in clinicopathological features (Figure 4(e)) between subgroups, marking the indicators with significant differences. The heat map (Figure 4(f)) suggested that the distribution of AJCC stage, grade, and T stage differed between the high- and low-risk groups and that the three lncRNAs were significantly upregulated in the high-risk patients.

### 3.4. Nomogram

**Figure 5:**
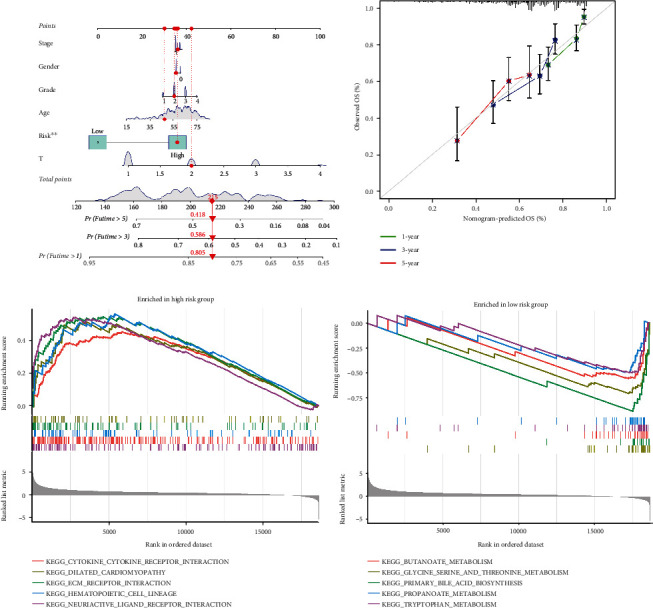
Nomogram and GSEA in TCGA cohort. (a) A nomogram for predicting the 1-, 3-, and 5-year OS of HCC patients in the TCGA cohort. (b) Calibration curves of the nomogram to predict the 1-, 3-, and 5-year OS of HCC patients in the TCGA cohort. (c) GSEA of high-risk group. (d) GSEA of low-risk group.

The nomogram was constructed based on clinical features and prognosis-related CRlncRNAs, and the calibration curve was close to the diagonal line, suggesting that the OS predicted by the nomogram was stable and accurate (Figures 5(a) and 5(b)).

### 3.5. Gene Set Enrichment Analysis

The annotated gene sets (“c2.cp.kegg.v7.4.symbols.gmt”) were downloaded from the Molecular Signatures Database (MSigDB, https://www.gsea-msigdb.org/gsea/msigdb). We extracted 5 signaling pathways from each of the two groups according to the *p* value ranking. The results showed that dilated cardiomyopathy, cytokine–cytokine receptor interactions, ECM-receptor interactions, neuroactive ligand–receptor interactions and hematopoietic cells were mainly enriched in the high-risk groups (Figure 5(c)). Butanoate metabolism, primary bile acid biosynthesis, glycine, serine and threonine metabolism, propanoate metabolism, and tryptophan metabolism were mainly enriched in the low-risk groups (Figure 5(d)).

### 3.6. Different Immune Landscapes and Gene Correlation Analysis in High- and Low-Risk Groups of HCC

**Figure 6:**
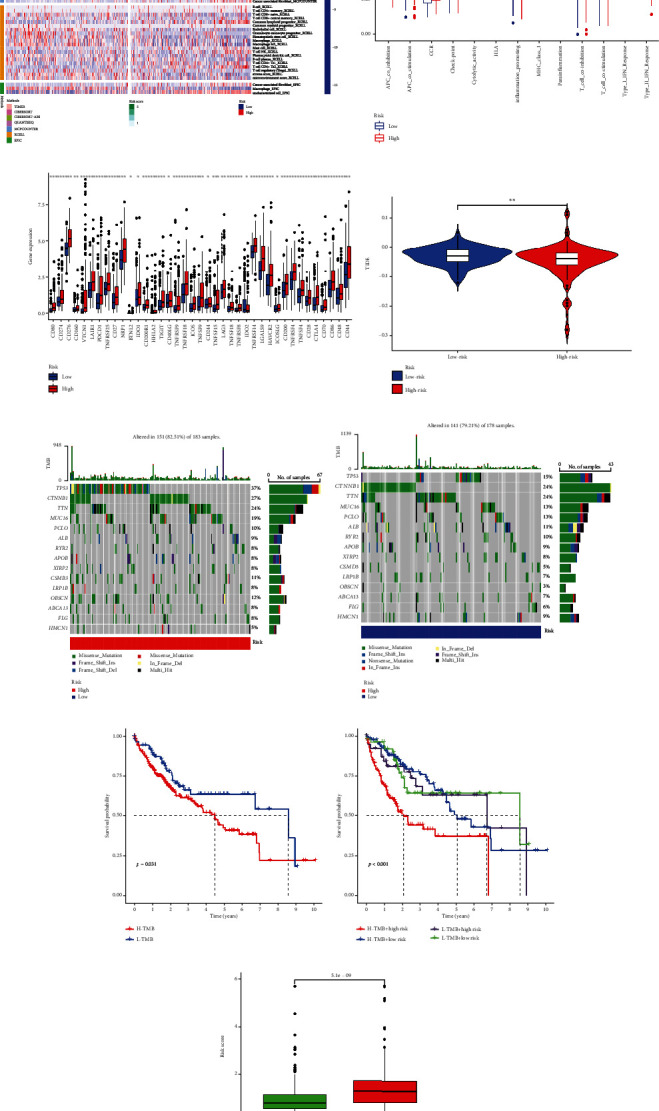
Immune landscape and tumor mutation burden (TMB) in TCGA cohort. (a) Analysis of immune infiltration based seven algorithms. Differential analysis of (b) immune function, (c) immune checkpoints and (d) TIDE scores. Waterfall diagram of the top 15 genes with the highest mutation frequency in (e) high- and (f) low-risk groups. (g) K-M survival analysis of the high- and low-risk group. (h) K-M survival analysis of combined TMB and risk scores. (i) Differential analysis of risk scores between the wild type and the mutation type of TP53.

The immune heat map was drawn based on seven algorithms including TIMER, CIBERSORT, CIBERSORT-ABS, QUANTISEQ, MCPCOUNTER, XCELL, and EPIC, and the relationship between prognosis and immunity in HCC patients was further evaluated (Figure 6(a)). The ssGSEA quantitative assessment of HCC showed that APC costimulation, CCR, cytolytic activity, MHC class I, Type I IFN response, and Type II IFN response were significantly different between the high- and low-risk groups (Figure 6(b)). Prior studies have shown that immune checkpoint blockade through epigenetic mechanisms is promising in HCC treatment and may determine prognosis. We evaluated the expression levels of immune checkpoint genes between the high- and low-risk groups (Figure 6(c)). Interestingly, in addition to IDO2, other immune checkpoints were highly expressed in the high-risk group. The TIDE algorithm revealed that patients in the low-risk group had a greater potential for immune escape (Figure 6(d)).

### 3.7. Tumor Mutational Burden

The importance of TMB in predicting the immune checkpoint blockade (ICB) response has been increasingly recognized, and tumors with a higher TMB tend to be more sensitive to ICB therapy [24]. The waterfall diagram demonstrated that for most genes, the mutation frequency was higher in the high-risk group than in the low-risk group. In addition, a waterfall diagram showed that the five most frequent somatic mutations in both the high-risk and low-risk groups were those in TP53, CTNNB1, TTN, MUC16, and PCLO (Figures 6(e) and 6(f)). Kaplan–Meier survival analysis revealed significant worse prognosis in the H-TMB and/or high-risk groups (Figures 6(g) and 6(h)). In addition, we showed the top 15 genes with the highest mutation counts in the TCGA cohort (Table 3). Furthermore, the risk scores between the wild type and the mutation type of TP53 were compared. We found that the risk scores in the mutation types of TP53 were significantly higher than that in the wild types (Figure 6(i)).

### 3.8. Externally Validation

**Figure 7:**
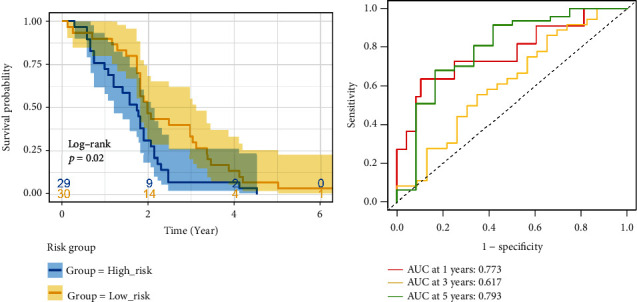
Validation of the signature in the external cohort: GSE40144. (a) K-M survival analysis. (b) ROC curve assessed 1-, 2-, and 3-year OS of HCC patients in the external validation cohort.

In the externally validated cohort, Kaplan–Meier analysis based on the median risk score of the TCGA cohort showed that the high-risk group had significantly lower OS than the low-risk group (*p* < 0.05), and ROC analysis showed that the signature when applied to the GEO cohort had higher predictive power (1 − year AUC = 0.773, 2 − year AUC = 0.617, and 3 − year AUC = 0.793; Figures 7(a) and 7(b)).

### 3.9. Validation of Expression Levels of Candidate Genes

**Figure 8:**
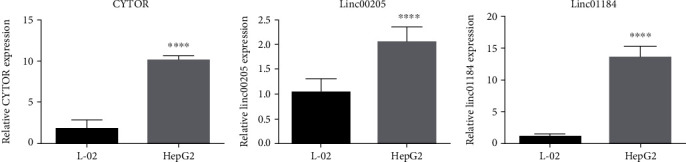
qRT-PCR analysis of 3 prognostic genes. (a) CYTOR. (b) LINC00205. (C) LINC01184. ^∗^*p* < 0.05, ^∗∗^*p* < 0.01, ^∗∗∗^*p* < 0.001, ^∗∗∗∗^*p* < 0.001.

We further validated the differential expression of 3 prognostic genes (CYTOR, LINC00205, LINC01184) between HCC cell lines and normal liver tissue samples. The qRT–PCR results showed that the expression levels of CYTOR, LINC00205, and LINC01184 were significantly upregulated in HepG2 cells compared to in LO2 cells (*p* < 0.0001), and the expression levels of these prognostic genes were consistent with the results of the bioinformatics analysis (Figure 8(a)–8(c)).

## 4. Discussion

In the past few decades, many copper enzymes, copper transporters, and copper chaperones have been discovered [25] as well as their biological functions. Elevated copper levels have been linked to a variety of cancers [26], including colorectal cancer [27], prostate cancer [28], breast cancer [29], pancreatic cancer [30], and cervical cancer [31]. The concept of cuproptosis was recently proposed and the mechanism of copper-induced cell death is protein lipidation, with the FDX1 and lipoic acid genes being critical mediators [1]. This discovery will provide new ideas for cancer treatment. Copper ionophores tend to overcome the limitations of traditional anticancer drugs by selectively inducing copper downregulation [32]. Inhibiting metabolic reprogramming on which cancer cells depend may be an effective way of limiting copper bioavailability.

In this study, we first identified 44 pairs of lncRNA-CRG coexpression relationships using Pearson correlation. Then we performed LASSO Cox regression analysis, obtained 3 prognosis-related lncRNAs, CYTOR, LINC00205, and LINC01184, and constructed a prognostic signature. The AUC values of this signature in the total set indicate that this signature has predictive ability. The reliability of the signature was verified by K-M, PCA, and C-index analysis, and it was demonstrated that these lncRNAs are potential prognostic markers and therapeutic targets for liver cancer. Previous studies have shown that CYTOR can promote liver cancer progression through regulation of the miRNA-125a-5p/LASP1 axis [33] and miR-125b/SEMA4C axis [34]. In addition, CYTOR was found to be a poor prognostic factor for gastric cancer, non-small-cell lung cancer, breast cancers, and nasopharyngeal carcinoma [35–38]. LINC00205 has been repeatedly proven to be involved in the progression of liver cancer through mutual regulation with miRNA and can be used as a biomarker for prognosis assessment and a potential target for disease diagnosis and treatment [39]. As in previous studies, LINC01184 effectively predicted the prognosis of HCC patients and promoted the progression of HCC [40].

Mutations in the TP53 tumor suppressor gene are among the most common genetic alterations in many human malignancies including liver cancer. In this study, missense mutations were the most common type of mutation, and TP53 mutations were the most frequently mutated gene, which can be identified in 96 HCC samples. It was found that the majority of TP53 mutations was missense and abundantly reported to be associated with poor prognosis in a variety of cancers [41].

Currently, immunotherapy plays an important role in the treatment of HCC as a promising new therapeutic strategy [42]. Notably, most immune checkpoint genes, including the well-known CTLA4 and PDCD1 (PD-1), were significantly upregulated in the high-risk group, suggesting the potential therapeutic targets for ICB applications. TIDE scores can predict patient response to immunotherapy, as they can reflect the potential capacity for the tumor's immune evasion. In our study, patients in the high-risk group had lower TIDE scores, which mean that they will benefit more from immunotherapy. By comparing tumor and normal liver tissue, we found that genes enriched in the following functional categories, including myeloid dendritic cells, memory B cells, CD4+ T cells, Tregs, T-cell follicular helper, neutrophils, M0 and M2 macrophages, resting/activated NKs, and resting mast cells were differentially expressed between the high- and low-risk groups. The infiltration of Tregs has been considered an important regulatory mechanism of immune system homeostasis and immune tolerance. Previous studies have shown that Tregs can secrete immunosuppressive cytokines, such as TGF-*β*, IL-10, and IL-35, and inhibit the antigen presentation functions of dendritic cells, which is an important reason for the upregulation of Tregs in high-risk patients [43].

## 5. Conclusion

A 3-CRlncRNA prognostic signature was constructed to predict clinical prognosis of HCC patients. A series of validations confirmed that the signature was stable and reliable. These results might be beneficial for individualized treatment and medical decision-making during the management of HCC patients. Although we have constructed a robust signature and validated it experimentally, the results still need to be treated with caution and need to be further verified in clinical practice.

## Figures and Tables

**Table 1:** Clinical characteristics of GEO database and three data sets randomly generated from the TCGA database.

Characteristics	TCGA-LIHC cohort			GSE40144 cohort
	Training set	Testing set	Total set	
	*n* = 185	*n* = 185	*n* = 370	
Age				
< = 65	122(66.0)	110(59.5)	232(62.7)	50 (84.7)
> 65	63(34.1)	75(40.5)	138(37.3)	9(15.3)
Gender				
Female	53(28.7)	68(36.8)	121(32.7)	12(20.3)
Male	127(71.4)	117(63.2)	249(67.3)	47(79.7)
AJCC stage				
I	81(43.8)	90(48.7)	171(46.2)	29(49.2)
II	40(21.6)	45(24.3)	85(23)	21(35.6)
III	43(23.2)	42(22.7)	85(23)	9(15.3)
IV	3(1.6)	2(1.1)	5(1.4)	0
T				
T1	88(47.6)	93(50.3)	181(48.9)	NA
T2	48(26.0)	45(24.3)	93(25.1)	NA
T3	41(22.2)	39(21.1)	80(21.6)	NA
T4	6(3.2)	7(3.8)	13(3.5)	NA
M				
M0	122(66.0)	144(77.8)	266(71.9)	NA
M1	2(1.1)	2(1.1)	4(1.1)	NA
N				
N0	116(62.7)	136(73.5)	252(68.1)	NA
N1	3(1.6)	1(0.5)	4(1.1)	NA

**Table 2:** Primer sequences for qRT–PCR.

Gene	Primer sequence (5′-3′)
CYTOR	Forward: CAGGTATCAGGCACAGCATCT
	Reverse: CAGGAAGCGTGAGGACAGAA
LINC00205	Forward: TTGAGACGGGAGTGTTCAGC
	Reverse: TCACTGGAGAGGGAGACGAG
LINC01184	Forward: GCAAGCGGTCTTCTCTGTCT
	Reverse: GTCTCCTGTTCGTGTCAGCA

**Table 3:** Gene mutation counts of samples in the TCGA cohort (Top 15).

Gene	Count
TP53	96
CTNNB1	94
TTN	89
MUC16	59
PCLO	41
ALB	39
RYR2	33
APOB	31
CSMD3	29
LRP1B	29
XIRP2	29
ABCA13	27
OBSCN	27
HMCN1	26
FLG	26

## Data Availability

The datasets presented in this study can be found in online repositories, further inquiries can be directed to the corresponding author.
